# Supplementary material for: A histone H3K4me1-specific binding protein is required for siRNA accumulation and DNA methylation at a subset of loci targeted by RNA-directed DNA methylation
Source: Nat Commun. 2021 Jun 7;12:3367. doi: 10.1038/s41467-021-23637-4 (PMC8184781; doi:10.1038/s41467-021-23637-4)
Supplement: Supplementary file 1 — Supplementary Information [file 41467_2021_23637_MOESM1_ESM.pdf]

Supplementary Table 1. Detailed list of the parameters measured by ITC.

Supplementary Table 2. Summary of X-Ray diffraction data and structure refinement statistics.

Supplementary Table 3. Primer information.

Supplementary Figures

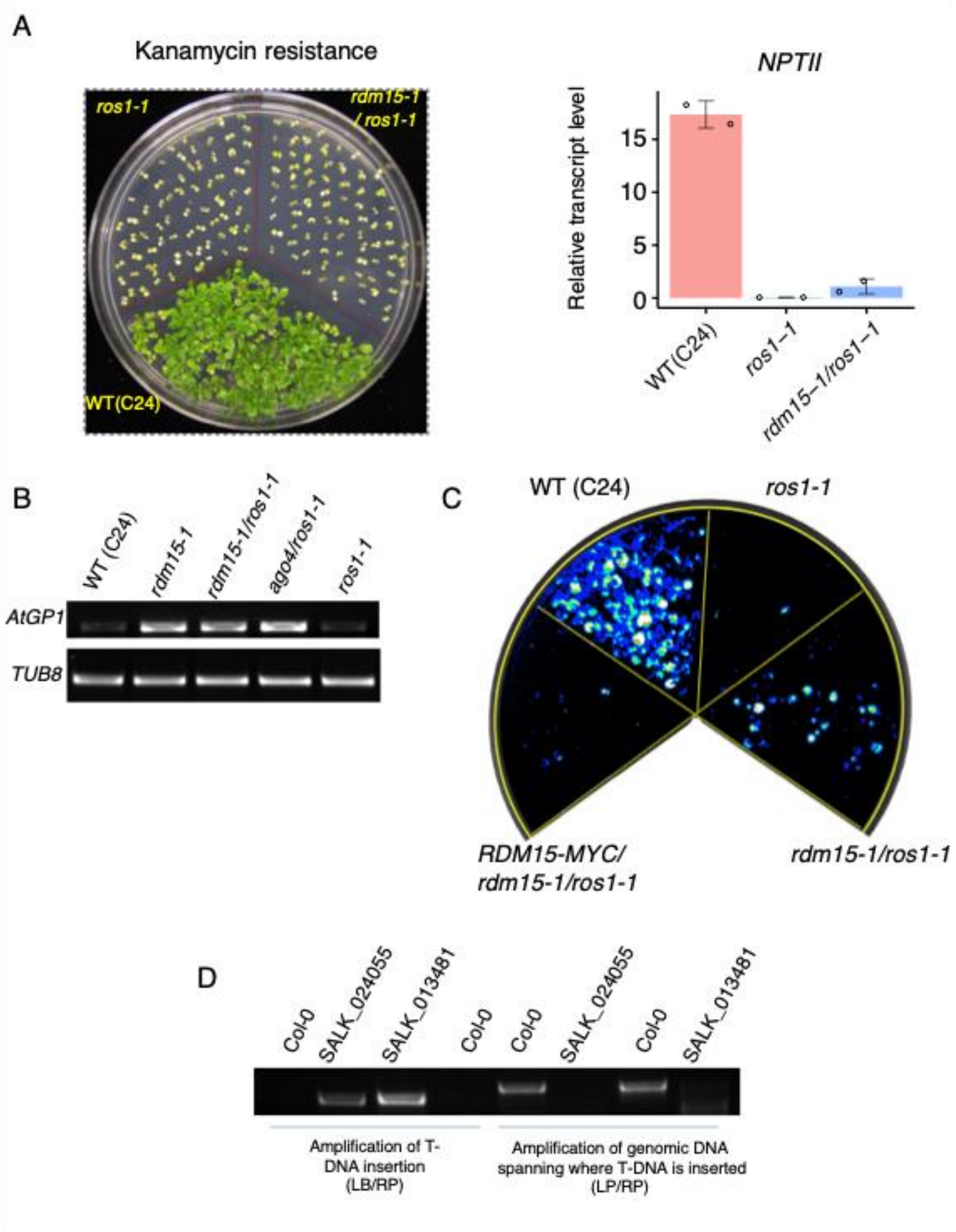

Supplementary Figure 1. RDM15 functions as a silencing factor in RdDM.

a. Effect of *rdm15-1* on the kanamycin-resistant phenotype in the *ros1-1* background. WT (C24) carries *35S-NPTII*, the expression of which leads to kanamycin-resistance. In the *ros1-1* background, the *rdm15* mutation failed to rescue the kanamycin-resistant phenotype (left) and *35S-NPTII* expression (right). Error bars represent s.d.

(n= 2 biologically independent samples). Data are presented as mean values +/- SD and the dots are the corresponding data points. **b.** RT-PCR analysis of transcript levels of endogenous *AtGP1,* which is a canonical RdDM target, in the WT (C24), *rdm15-1*, *rdm15-1/ros1-1*, *ago4/ros1-1*, and *ros1-1* genotypes. Error bars represent s.d. (n=2). Data are presented as mean values +/- SD and the dots are the corresponding data points. **c**. Complementation of *rdm15-1/ros1-1* by *RDM15-MYC*. The WT, *ros1-1*, *rdm15-1ros1-1*, and *RDM15-MYC*/*rdm15-1ros1-1* plants were grown for 10 days and imaged after cold treatment (48 h, 4°C). Compared to *rdm15-1ros1-1*, *RDM15-MYC*/*rdm15-1ros1-1* showed weaker luminescence emission.
d. *rdm15-2* and *rdm15-3* plants containing T-DNA insertions were confirmed by PCR amplification and agarose gel electrophoresis.

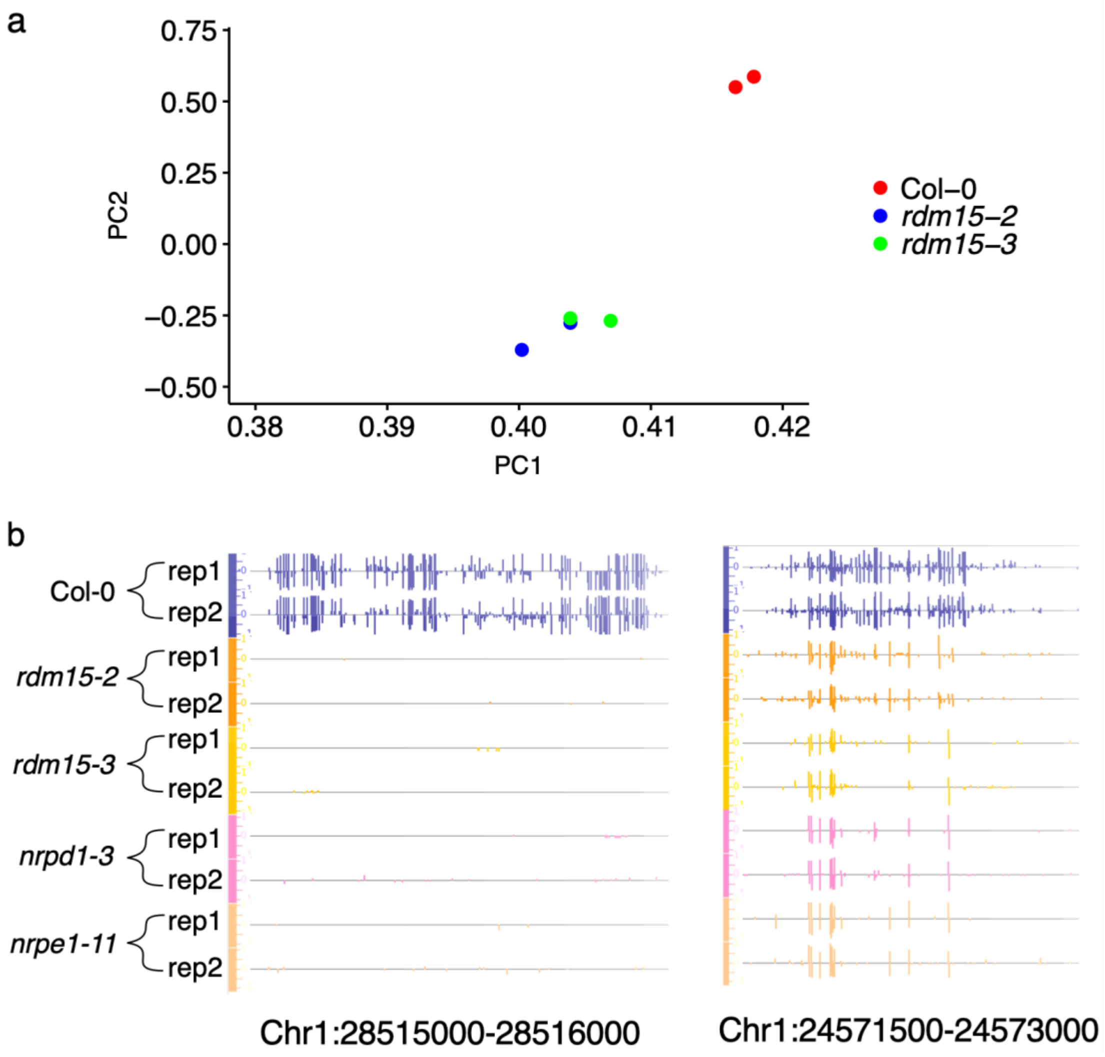

Supplementary Figure 2. Effect of *rdm15* on genomic cytosine methylation.

a. Principal components analysis of DNA methylomes of Col-0, *rdm15-2*, and *rdm15-3* with two biological replicates. **b**. DNA methylation levels of representative *rdm15* DMRs in Col-0, *rdm15-2*, *rdm15-3*, *nrpd1-3*, and *nrpe1-11*.

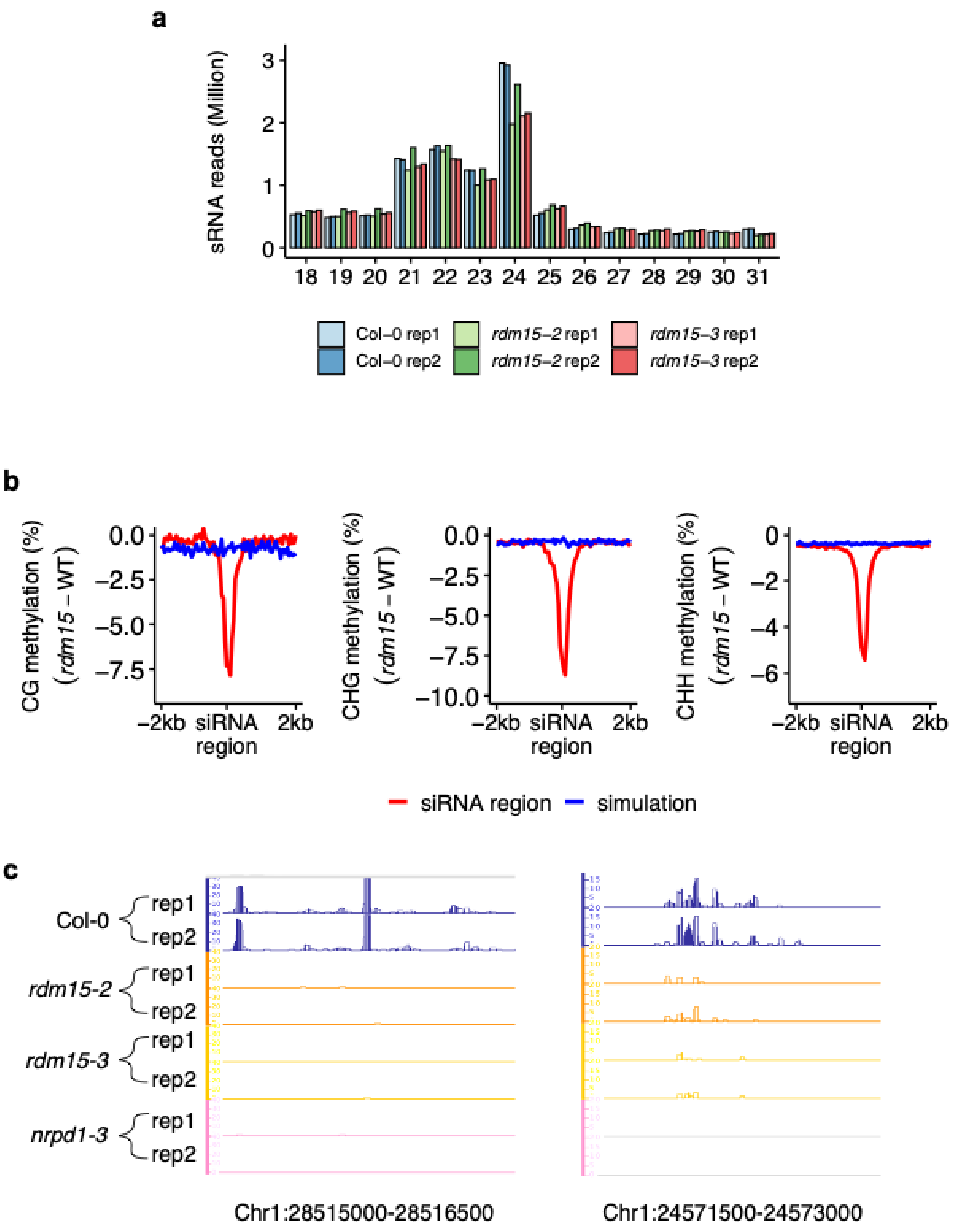

Supplementary Figure 3. Effect of *rdm15* on the accumulation of siRNAs.

a. Genome-wide siRNA profiles of Col-0, *rdm15-2*, and *rdm15-3* with two biological replicates. **b**. Change of CG CHG and CHH methylation levels (*rdm15*-WT) at RDM15-dependent siRNA regions. Control regions were randomly selected regions that were simulated with the lengths of RDM15-dependent siRNA regions. **c**. IGB display of the siRNA levels at several *rdm15* hypo-DMRs.

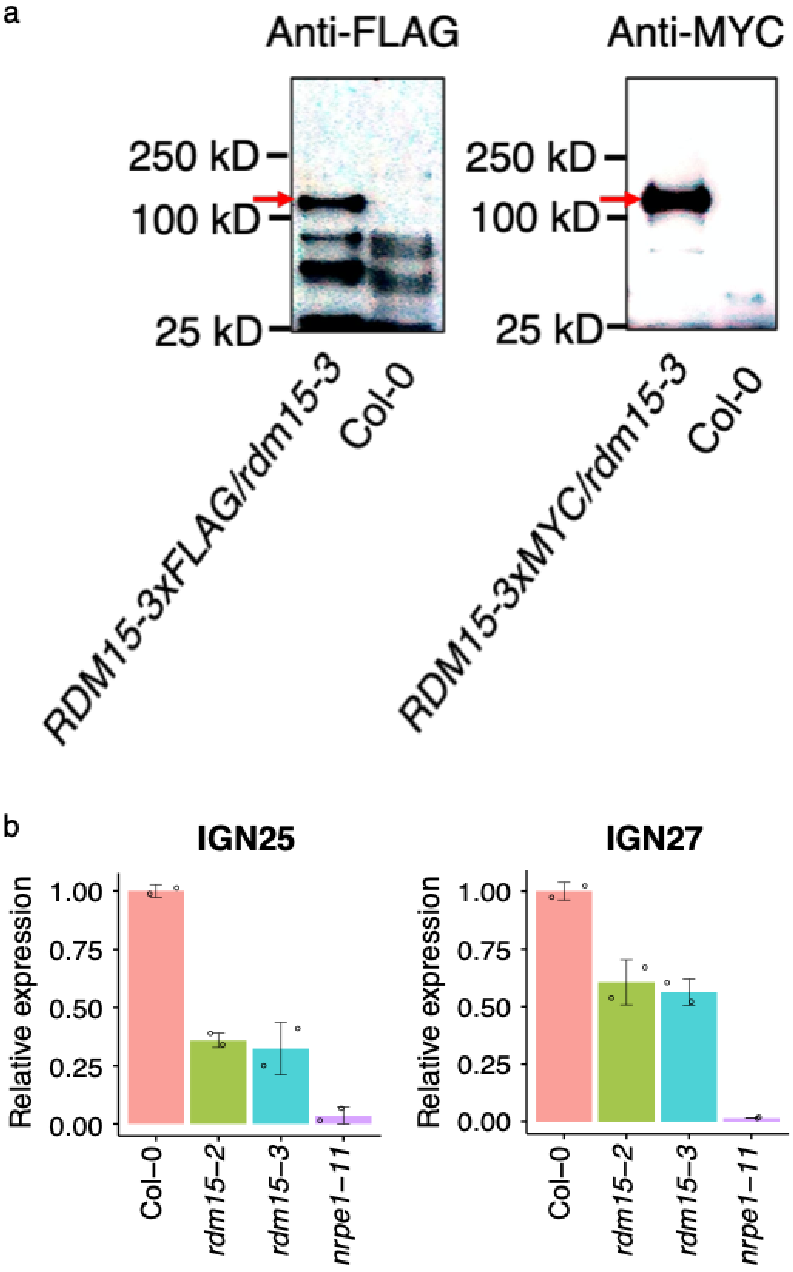

Supplementary Figure 4. Relationship between RDM15 and Pol V-dependent transcription. a. Western blot showing RDM15 protein accumulation in *RDM15-3MYC/rdm15-3* and *RDM15-3FLAG/rdm15-3* plants. **b.** RT-qPCR analysis of relative transcript levels of Pol V-dependent transcript *IGN25* and *IGN27* in Col-0 (WT), *rdm15-2*, *rdm15-3*, and *nrpe1-11*. Error bars represent s.d. (n=2 biologically independent samples). Data are presented as mean values +/- SD and the dots are the corresponding data points.

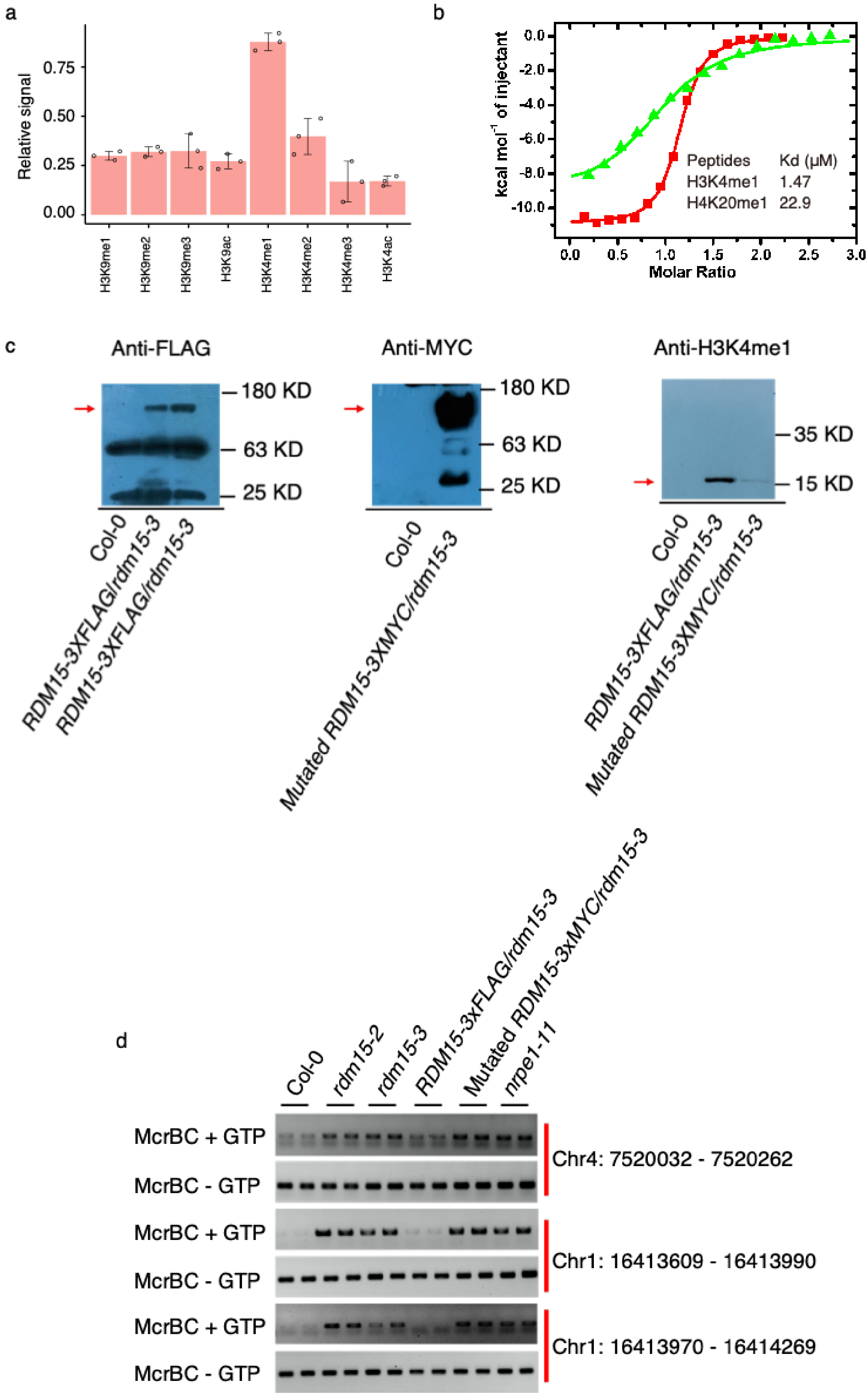

Supplementary Figure 5. The RDM15 tudor domain specifically recognizes the

H3K4me1 mark. a. Histone peptide array assay showing the binding preference of RDM15 to H3K4me1. Error bar represents s.d. (n=3 independent experiments). Data are presented as mean values +/- SD and the dots are the corresponding data points. **b.** ITC binding curves between the RDM15 tudor domain and H3K4me1 and H4K20me1 peptides indicate that H3K4me1 is the optimal binding partner for RDM15. **c.** Western blots showing that H3K4me1 was co-immunoprecipitated by RDM15 but not by mutated RDM15. The *RDM15-3xFLAG/rdm15-3* and *mutated RDM15-3xMYC/rdm15-3* plants were used to perform protein immunoprecipitation with anti-FLAG and anti-MYC antibodies, respectively, and then anti-H3K4me1 antibody was used to detect H3K4me1. **d.** Methylation specific PCR results showing the methylation levels at several RDM15-dependent RdDM loci. McrBC, an enzyme cleaving methylated DNA, was used to digest genomic DNA from different plants with two biological replicates. Stronger PCR signal represents lower methylation level. *RDM15-3xFLAG* rescued the hypomethylation phenotype in *rdm15-3*, however, the Mutated *RDM15-3xMYC* didn't rescue the methylation phenotype.

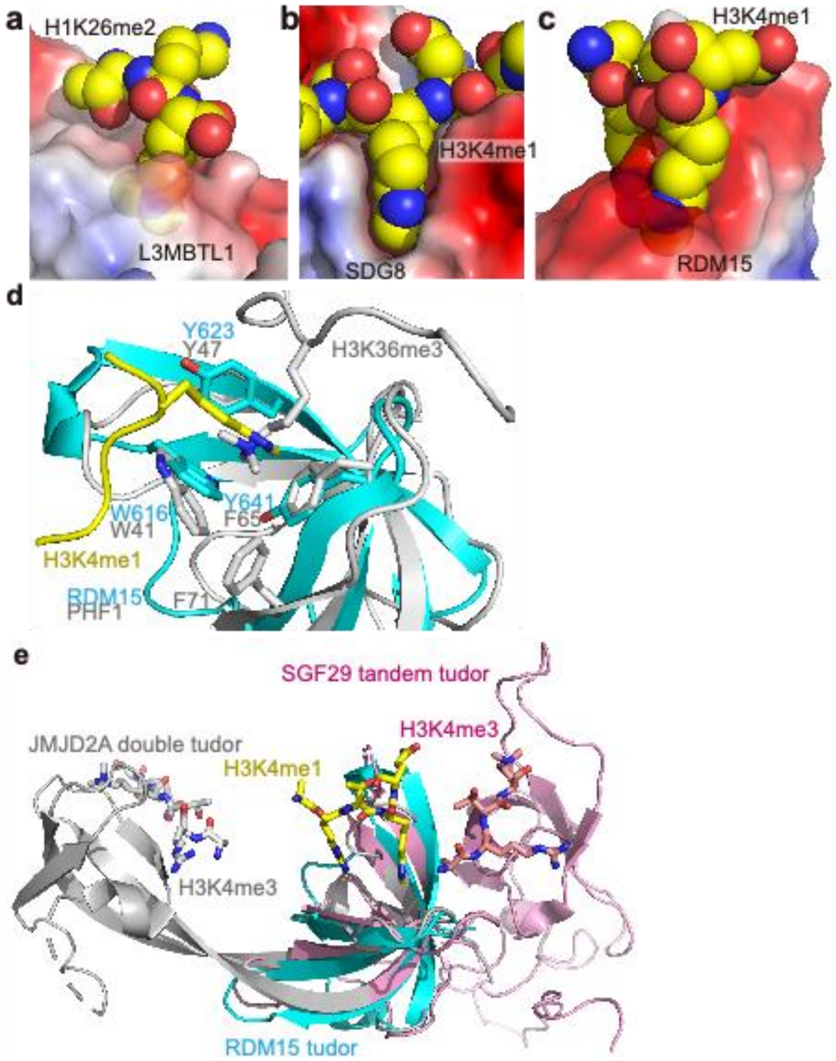

Supplementary Figure 6. The RDM15 tudor domain enables the recognition of lower methyllysine. a-c. Structures of the L3MBTL1-H1K26me2 complex (a, PDB code: 2RHI), SDG8-H3K4me1 complex (b, PDB code: 5YVX), and RDM15-

H3K4me1 complex (c, in this study), with reader proteins in electrostatics surface view and histone peptide in space-filling representation. L3MBTL1 and SDG8 use deep surface cavities to specifically recognize the lower methylation states of methyllysine; in these interactions, the side chains of the methyllysines are almost fully buried in a cavity of the protein, and this serves as a physical shape-based size selection filter to control methylation status. RDM15 uses a surface groove pocket to recognize methyllysine. The RDM15 tudor domain can form hydrogen bonds with two monomethylammonium protons of monomethyllysine to enhance the binding affinity, so as to obtain the selectivity for lower methylation state. **d.** Superposition of RDM15-H3K4me1 (in cyan and yellow) and PHF1-H3K36me3 (in silver, PDB code: 4HCZ) complexes reveals that although they have a similar aromatic cage, the peptides adopt opposite orientations. PHF1 has a broader aromatic cage, which is capable of recognizing higher methylation states. In contrast, RDM15 has a narrow cage, which is suitable for recognizing lower methylation states. **e.** Superimposition of RDM15-H3K4me1 (in cyan and yellow) and JMJD2A double tudor-H3K4me3 complex (PDB code: 2GFA, in silver) and SGF29 tandem tudor-H3K4me3 complex (PDB code: 3MEA, in light magenta) reveal that they have distinct histone mark binding modes.

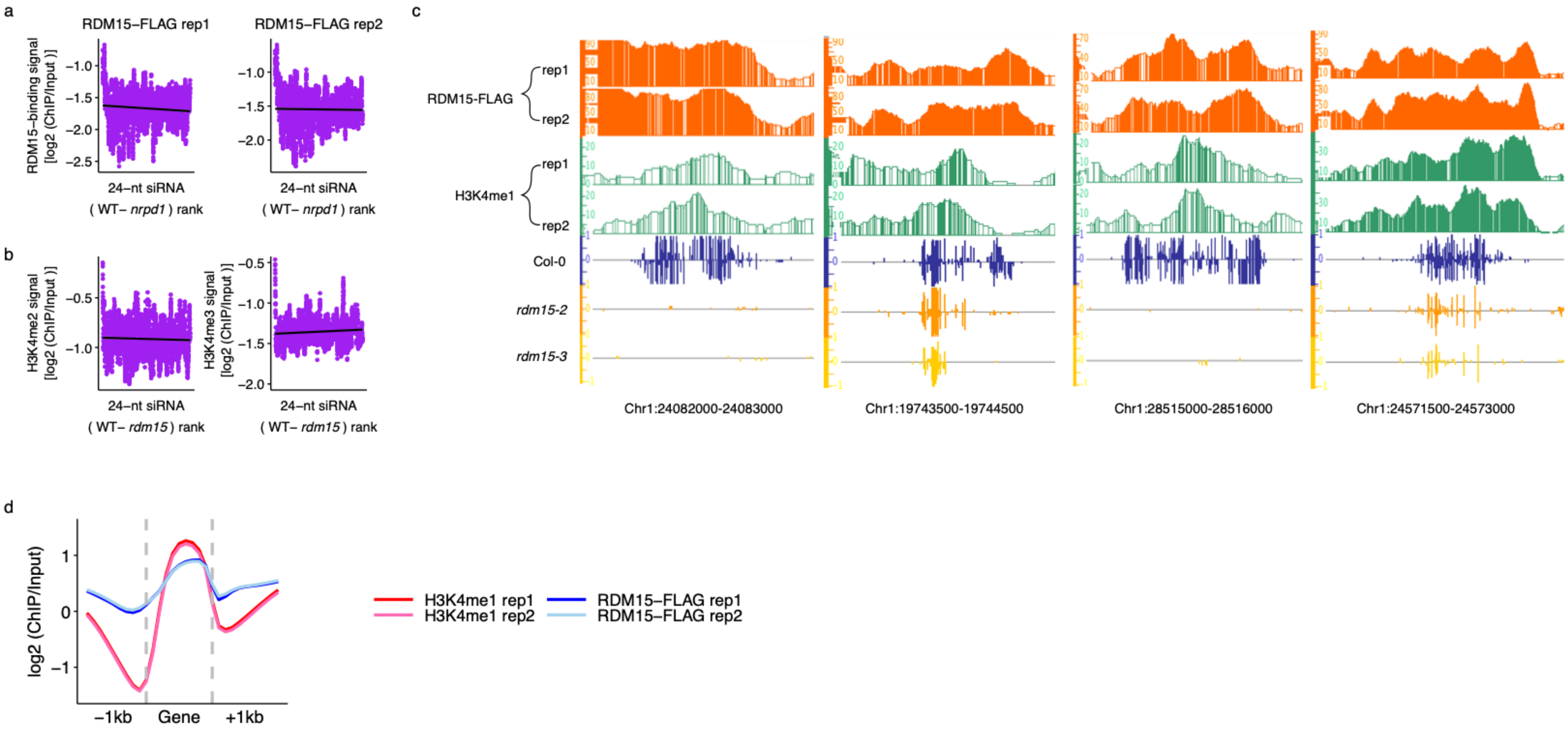
a
RDM15–FLAG rep1
RDM15–FLAG rep2
RDM15–binding signal [log2 (ChIP/Input)]
24–nt siRNA (WT– nrpd1) rank
b
H3K4me2 signal [log2 (ChIP/Input)]
H3K4me3 signal [log2 (ChIP/Input)]
24–nt siRNA (WT– rdm15) rank
c
RDM15-FLAG
H3K4me1
rep1
rep2
Col-0
rdm15-2
rdm15-3
Chr1:24082000-24083000
Chr1:19743500-19744500
Chr1:28515000-28516000
Chr1:24571500-24573000
d
log2 (ChIP/Input)
−1kb
Gene
+1kb
H3K4me1 rep1
H3K4me1 rep2
RDM15–FLAG rep1
RDM15–FLAG rep2

Supplementary Figure 7. Enrichment of RDM15 at RDM15-targeted regions. a. Change of siRNAs (WT vs. *nrpd1*) at Pol IV-only siRNA regions was not correlated with RDM15 enrichment. **b.** Change of siRNAs (WT vs. *rdm15*) at RDM15-dependent siRNA regions was not correlated with H3K4me2 or H3K4me3 enrichment. **c.** RDM15 and H3K4me1 enrichment and DNA methylation levels of several RDM15-targeted regions. **d.** Metaplot profiles showing average ChIP signals of H3K4me1 and RDM15-3xFLAG around genes.

Supplementary Table 1. Detailed list of the parameters measured by ITC.

Protein	Peptide	N Value	Kd (μM)	ΔH (kcal/mol)	ΔS (cal/mol/deg)
Wild-type	H3K4me1	0.96±0.03	1.47±0.17	-9.34±0.50	-9.63
Wild-type	H3K4me2	1.03±0.04	30.9±4.67	-8.89±0.62	-10.3
Wild-type	H3K4me3	0.90±0.07	66.2±25.4	-3.83±0.40	4.81
Wild-type	H3K4me0	-	NDB	-	-
Y623A	H3K4me1	-	NDB	-	-
W616A	H3K4me1	-	NDB	-	-
Y641A	H3K4me1	-	NDB	-	-
D643A	H3K4me1	-	NDB	-	-
D645A	H3K4me1	0.86±0.20	62.7±12.8	-3.51±1.14	6.95
E647A	H3K4me1	-	NDB	-	-
Q654A	H3K4me1	1.08±0.03	60.8±22.8	-6.07±0.25	1.62
Wild-type	H4K20me1	0.96±0.04	22.9±4.31	-9.23±0.53	-9.16

Buffer: 100mM NaCl, 20mM HEPES [7.4], 2mM β-mercaptoethanol. NDB, no detectable binding.

Supplementary Table 2. Diffraction Data and Structure Refinement Statistic

	Se-RDM15-H3K4me1
**Data collection**	
Space group	$P2_12_12_1$
Cell dimensions	
a, b, c (Å)	28.6, 37.0, 46.7
α, β, γ (°)	90, 90, 90
Resolution (Å)	50.0-1.7 (1.76-1.70)*
R_{sym} or R_{merge}	0.099 (0.500)
$I / \sigma I$	26.7 (4.8)
Completeness (%)	99.9 (99.8)
Redundancy	8.7 (9.0)
**Refinement**	
R_{work} / R_{free}	0.183 / 0.202
No. atoms	542
Protein	475
Peptide	42
Water	25
B-factors (Å^2)	32.7
Protein	32.4
Peptide	32.6
Water	37.9
R.m.s. deviations	
Bond lengths (Å)	0.005
Bond angles (°)	0.954

*Highest-resolution shell is shown in parentheses.

Supplementary Table 3. Primer information.

**Primers**	
RDM15-nLUC-F	ACGGGGGACGAGCTCGGTACCATGTCGGATTCTGATAAAGAGCTC
RDM15-nLUC-R	CGCGTACGAGATCTGGTCGACTCGCTTCCTCTTCTTACCGGACTT
RDM15-cLUC-F	TACGCGTCCCGGGGCGGTACCATGTCGGATTCTGATAAAGAGCTC
RDM15-cLUC-R	ACGAAAGCTCTGCAGGTCGACTCGCTTCCTCTTCTTACCGGACTT
NRPE3B-nLUC-F	ACGGGGGACGAGCTCGGTACCATGGACGGTGTCACCTACCAAAGA
NRPE3B-nLUC-R	CGCGTACGAGATCTGGTCGACTCCTTCACGCATATGGGCACCGAG
NRPE3B-cLUC-F	TACGCGTCCCGGGGCGGTACCATGGACGGTGTCACCTACCAAAGA
NRPE3B-cLUC-R	ACGAAAGCTCTGCAGGTCGACTCCTTCACGCATATGGGCACCGAG
RDM15-YN-F	ATTTACGAACGATAGTTAATTAACATGTCGGATTCTGATAAAGAGCTC
RDM15-YN-R	ACTGCCACCTCCTCCACTAGTTCGCTTCCTCTTCTTACCGGACTT
RDM15-YC-F	ATTTACGAACGATAGTTAATTAACATGTCGGATTCTGATAAAGAGCTC
RDM15-YC-R	ACTGCCACCTCCTCCACTAGTTCGCTTCCTCTTCTTACCGGACTT
NRPE3B-YN-F	ATTTACGAACGATAGTTAATTAACATGGACGGTGTCACCTACCAAAGA
NRPE3B-YN-R	ACTGCCACCTCCTCCACTAGTTCCTTCACGCATATGGGCACCGAG
NRPE3B-YC-F	ATTTACGAACGATAGTTAATTAACATGGACGGTGTCACCTACCAAAGA
NRPE3B-YC-R	ACTGCCACCTCCTCCACTAGTTCCTTCACGCATATGGGCACCGAG
RDM15-3*FLAG-F	GCTATGACATGATACGAATTCGCTCTTCACATACACCGACACCTA
RDM15-3*FLAG-R	ATGGTCTTTGTAGTCAAGCTTTCGCTTCCTCTTCTTACCGGACTT
RDM15-3*MYC-F	CTATGACATGATTACGAATTCGCTCTTCACATACACCGACACCTA
RDM15-3*MYC-R	AGTCCATGGAGATCTAAGCTTTCGCTTCCTCTTCTTACCGGACTT
Chr4: [7520032 - 7520262]-F	GGGACTTGTCTCCCACAAAA
Chr4: [7520032 - 7520262]-R	CCATCAAACAGCCCATTGTA
Chr1: [16413609 - 16413990] –F	TCAGAAGCTAGCATGCTCCA
Chr1: [16413609 - 16413990] –R	AGCTAACTCCTGCATCAACAAA
Chr1: [16413970 - 16414269] –F	TGTTGATGCAGGAGTTAGCTTT
Chr1: [16413970 - 16414269] –R	CGGCAAGCTTCTTGATTTGT
**qPCR primers**	
ROS1-qF	AAGGACCAACTTGTTGCGAC
ROS1-qR	AGGACTCTATTAGCACTGAGC
LUC-qF	GGAGAGCAACTGCATAAGGCTATG
LUC-qR	GACGATTCTGTGATTTGTATTCAGC
RD29A-qF	CCTGTCGTGTCTTCCGATTACAC
RD29A-qF	GACAATTCGGACAGAGGATGATG
NPTII-qF	CAAGCTCTTCAGCAATATCACGG
NPTII-qR	GATGCCTGCTTGCCGAATATC
NRPD1-qF	CAAGGTACTCGATGGGAAAGG
NRPD1-qR	TGATCTCGTAACTGTGGAGAATG
NRPE1-qF	GTCCCAGACGCAGACATAAA
NRPE1-qR	CGCCATTCTCTCCACTCTTT
ACT2-qF2	TGTGTGACAAACTCTCTGGG
ACT2-qR2	GGCATCAATTCGATCACTCAG
TUB8-qF	AGCTGAGAATTGTGACTGCTTG
TUB8-qR	TCAACAACGTTCCCATTCC
ATGP1-qF	ACAGTGCCACAGTTGAGCAG
ATGP1-qR	CAGAAAAATACTCGGTGCCAAT
MEA-ISR-qF	CGCGAACGACTATTGCTAAA
MEA-ISR-qR	TGAAATCTAACCGGATTTTGG
