# Supplementary material for: A histone H3K4me1-specific binding protein is required for siRNA accumulation and DNA methylation at a subset of loci targeted by RNA-directed DNA methylation
Source: Nat Commun. 2021 Jun 7;12:3367. doi: 10.1038/s41467-021-23637-4 (PMC8184781; doi:10.1038/s41467-021-23637-4)
Supplement: Supplementary file 2 — Reporting Summary [file 41467_2021_23637_MOESM2_ESM.pdf]

nature research

Corresponding author(s): Zhaobo Lang

Last updated by author(s): Mar 10, 2021

Reporting Summary

Nature Research wishes to improve the reproducibility of the work that we publish. This form provides structure for consistency and transparency in reporting. For further information on Nature Research policies, see our Editorial Policies and the Editorial Policy Checklist.

Statistics

For all statistical analyses, confirm that the following items are present in the figure legend, table legend, main text, or Methods section.

n/a	Confirmed	
☐	☒	The exact sample size (*n*) for each experimental group/condition, given as a discrete number and unit of measurement
☐	☒	A statement on whether measurements were taken from distinct samples or whether the same sample was measured repeatedly
☐	☒	The statistical test(s) used AND whether they are one- or two-sided<br>*Only common tests should be described solely by name; describe more complex techniques in the Methods section.*
☒	☐	A description of all covariates tested
☐	☒	A description of any assumptions or corrections, such as tests of normality and adjustment for multiple comparisons
☐	☒	A full description of the statistical parameters including central tendency (e.g. means) or other basic estimates (e.g. regression coefficient) AND variation (e.g. standard deviation) or associated estimates of uncertainty (e.g. confidence intervals)
☒	☐	For null hypothesis testing, the test statistic (e.g. *F*, *t*, *r*) with confidence intervals, effect sizes, degrees of freedom and *P* value noted<br>*Give P values as exact values whenever suitable.*
☒	☐	For Bayesian analysis, information on the choice of priors and Markov chain Monte Carlo settings
☒	☐	For hierarchical and complex designs, identification of the appropriate level for tests and full reporting of outcomes
☒	☐	Estimates of effect sizes (e.g. Cohen's *d*, Pearson's *r*), indicating how they were calculated

Our web collection on statistics for biologists contains articles on many of the points above.

Software and code

Policy information about availability of computer code

Data collection	We created an Bisulfite-seq, sRNA and ChIP-seq library and sequenced it on the HiSeq2500.<br>All the diffraction data were collected at the Shanghai Synchrotron Radiation Facility beamline BL17U1 and were processed using the HKL2000 program.
Data analysis	For ITC analysis: ITC binding was measured using a Microcal PEAQ-ITC instrument (Malvern).The data were analyzed using the Origin 7.0 program;<br>For ChIP-Seq: The paired-end reads were mapped to the TAIR10 genome of Arabidopsis thaliana (TAIR10) with bowtie2. To remove potential PCR duplicates, markdup from SAMtools-1.8 was used. After mapping, only uniquely mapped reads were retained for downstream analysis. Fragment number of interested regions was counted by featureCounts v1.4.6. The public data for H3K4me2 and H3K4me3 were downloaded from the NCBI GEO database with accession number GSE113076.

For manuscripts utilizing custom algorithms or software that are central to the research but not yet described in published literature, software must be made available to editors and reviewers. We strongly encourage code deposition in a community repository (e.g. GitHub). See the Nature Research guidelines for submitting code & software for further information.

Data

Policy information about availability of data

All manuscripts must include a data availability statement. This statement should provide the following information, where applicable:

- Accession codes, unique identifiers, or web links for publicly available datasets
- A list of figures that have associated raw data
- A description of any restrictions on data availability

The data discussed in this publication have been deposited in NCBI's Gene Expression Omnibus and are accessible through GEO Series accession number

GSE154302(https://www.ncbi.nlm.nih.gov/geo/query/acc.cgi?acc=GSE154302). The structure of the Arabidopsis RDM15 tudor domain in complex with an H3K4me1 peptide has been deposited into the RCSB Protein Data Bank with the accession code of 7DE9 [https://www.rcsb.org/structure/7DE9].

Field-specific reporting

Please select the one below that is the best fit for your research. If you are not sure, read the appropriate sections before making your selection.

☒ Life sciences ☐ Behavioural & social sciences ☐ Ecological, evolutionary & environmental sciences

For a reference copy of the document with all sections, see nature.com/documents/nr-reporting-summary-flat.pdf

Life sciences study design

All studies must disclose on these points even when the disclosure is negative.

Sample size	Gene expression and BS-seq and RNA-seq: at least 15 plants were selected for each genotype.
Data exclusions	No data was excluded.
Replication	For RNA-seq and qRT-PCR analysis, we take three biological replications to verify the reproducibility of the experiment findings. For BS-seq, we take two biological replications to verify the reproducibility of the experiment findings.
Randomization	ChIP-seq samples and IP-MS samples were grown in same environmental conditions. we used randomization to allocate different samples.
Blinding	For all experiments, we analyzed the results in seedings of different mutants and therefore blinding was not possible for us.

Reporting for specific materials, systems and methods

We require information from authors about some types of materials, experimental systems and methods used in many studies. Here, indicate whether each material, system or method listed is relevant to your study. If you are not sure if a list item applies to your research, read the appropriate section before selecting a response.

Materials & experimental systems

n/a	Involved in the study	
☐	☒	Antibodies
☒	☐	Eukaryotic cell lines
☒	☐	Palaeontology and archaeology
☒	☐	Animals and other organisms
☒	☐	Human research participants
☒	☐	Clinical data
☒	☐	Dual use research of concern

Methods

n/a	Involved in the study	
☐	☒	ChIP-seq
☒	☐	Flow cytometry
☒	☐	MRI-based neuroimaging

Antibodies

Antibodies used	anti-GST (Abmart,#12G8);anti-flag (Sigma-Aldrich, #F3165); anti-myc (Sigma-Aldrich, #M5546); secondary antibody-Goat Anti-Mouse (M21001S, Abmart)
Validation	The specificity of antibodies used in this paper was extensively examined by the vendor and independent investigators. In addition, non-transgenic background-wild type was included for the validation of western analysis.<br>anti-GST (Abmart,#12G8):http://www.ab-mart.com.cn/upload/20180315105109xz.pdf<br>anti-flag (Sigma-Aldrich, #F3165):https://www.sigmaaldrich.com/catalog/product/sigma/f3165?lang=zh&region=CN<br>anti-myc (Sigma-Aldrich, #M5546):https://www.sigmaaldrich.com/catalog/product/sigma/m5546?lang=zh&region=CN;<br>econdary antibody-Goat Anti-Mouse (M21001S, Abmart):http://www.ab-mart.com.cn/page.aspx?node=62&id=17687

ChIP-seq

Data deposition

☒ Confirm that both raw and final processed data have been deposited in a public database such as GEO.

☒ Confirm that you have deposited or provided access to graph files (e.g. BED files) for the called peaks.

Data access links<br>*May remain private before publication.*	https://www.ncbi.nlm.nih.gov/geo/query/acc.cgi?acc=GSE154302

Files in database submission	The data discussed in this publication have been deposited in NCBI's Gene Expression Omnibus and are accessible through GEO Series accession number GSE154302
Genome browser session (e.g. UCSC)	Not applicable for this study

Methodology

Replicates	For RDM15 ChIP-seq, two biological replicates from a single-locus transgenic line expressing RDM15-FLAG are used.
Sequencing depth	RDM15_Flag_rep1 139395683 paired reads; RDM15_Flag_rep2 145669074 paired reads
Antibodies	anti-Flag M2 (Sigma-Aldrich, #F3165)(https://www.sigmaaldrich.com/catalog/product/sigma/f3165?lang=zh&region=CN)
Peak calling parameters	NA
Data quality	NA
Software	he paired-end reads were mapped to the TAIR10 genome of Arabidopsis thaliana (TAIR10) with bowtie2. To remove potential PCR duplicates, markdup from SAMtools-1.8 was used. After mapping, only uniquely mapped reads were retained for downstream analysis. Fragment number of interested regions was counted by featureCounts.
