# Supplementary material for: A histone H3K4me1-specific binding protein is required for siRNA accumulation and DNA methylation at a subset of loci targeted by RNA-directed DNA methylation
Source: Nat Commun. 2021 Jun 7;12:3367. doi: 10.1038/s41467-021-23637-4 (PMC8184781; doi:10.1038/s41467-021-23637-4)
Supplement: Supplementary file 4 — Description of Additional Supplementary Files [file 41467_2021_23637_MOESM4_ESM.pdf]

Description of Additional Supplementary Files

File name: Supplementary Data 1
Description: List of DMRs in different mutants.

File name: Supplementary Data 2
Description: List of RDM15-dependent siRNA cluster regions.

File name: Supplementary Data 3
Description: RDM15 IP-MS/MS.

File name: Supplementary Data 4
Description: Data of RDM15 histone peptide array assay.
